# Intercellular protein–protein interactions at synapses

**DOI:** 10.1007/s13238-014-0054-z

**Published:** 2014-04-23

**Authors:** Xiaofei Yang, Dongmei Hou, Wei Jiang, Chen Zhang

**Affiliations:** 1 Key Laboratory of Cognitive Science, Laboratory of Membrane Ion Channels and Medicine, College of Biomedical Engineering, South-Central University for Nationalities, Wuhan, 430074 China; 2 State Key Laboratory of Biomembrane and Membrane Biotechnology, College of Life Sciences, Peking University, Beijing, 100871 China; 3 PKU-IDG/McGovern Institute for Brain Research, Peking University, Beijing, 100871 China

**Keywords:** synapse formation, cell-cell adhesion, synaptic plasticity, neurological disorders, protein-protein interaction, cell adhesion molecules

## Abstract

Chemical synapses are asymmetric intercellular junctions through which neurons send nerve impulses to communicate with other neurons or excitable cells. The appropriate formation of synapses, both spatially and temporally, is essential for brain function and depends on the intercellular protein-protein interactions of cell adhesion molecules (CAMs) at synaptic clefts. The CAM proteins link pre- and post-synaptic sites, and play essential roles in promoting synapse formation and maturation, maintaining synapse number and type, accumulating neurotransmitter receptors and ion channels, controlling neuronal differentiation, and even regulating synaptic plasticity directly. Alteration of the interactions of CAMs leads to structural and functional impairments, which results in many neurological disorders, such as autism, Alzheimer’s disease and schizophrenia. Therefore, it is crucial to understand the functions of CAMs during development and in the mature neural system, as well as in the pathogenesis of some neurological disorders. Here, we review the function of the major classes of CAMs, and how dysfunction of CAMs relates to several neurological disorders.

## Introduction

The brain is characterized by an enormous degree of complexity and diversity of neural networks, making it one of the most complicated organs. This complexity and diversity come from the vast numbers of neurons, but also from the variety of synapses where neurons pass electrical or chemical signals to other cells. Most of the synapses are small in size (1 μm in diameter), but biochemical studies reveal a high level of the molecular complexity. The post-synaptic proteome of the excitatory synapse of a mammalian brain contains more than 1000 proteins, indicating complicated protein-protein interactions occurring both within and between synapses (Collins et al., 2006; Cheng et al., 2006; Peng et al., 2004; Bayés et al., 2011; Dosemeci et al., 2006; Fernández et al., 2009; Trinidad et al., 2008; Hahn et al., 2009; Satoh et al., 2002).

The synapse is the site where two neurons connect, separated by a narrow (~20 nm) layer of extracellular space called a synaptic cleft (Akert et al., 1972). The molecular composition of the synaptic cleft still remains largely unclear, but early studies demonstrated the presence of mostly proteins and carbohydrates (GRAY 1959; Pfenninger, 1971; Bloom and Aghajanian, 1966). One of the obvious functions of protein-protein interactions in the clefts is to serve as the “glue” that connects the pre- and post-synaptic neurons. Mounting evidence has confirmed that the adhesiveness of the pre- and post-synaptic compartment (for example, the attachment of pre- and post-synaptic membranes in a synaptosome preparation) is resistant to treatments such as calcium removal, high salt, or even low concentrations of urea treatment (Pfenninger, 1971; Cotman and Taylor, 1972). Further studies have revealed that the CAM-mediated intercellular protein-protein interactions at synapses, are involved in the recognition and alignment of pre- and post-synaptic sites, trans-synaptic signaling, and the precise localization of neurotransmitter receptors and releasable vesicles. Alterations in CAMs lead to changes in synaptic morphology and function, and are associated with many neurological disorders including autism, AD and schizophrenia. Several families of CAMs are recognized, including neurexins and neuroligins, leucine-rich repeat transmembrane neuronal proteins (LRRTMs), N-cadherin/β-catenin, ephrins and Eph receptors, SynCAM, and integrins. In this review we summarize the known CAMs, their physiology, and their roles in brain pathologies involving protein-protein interactions at synapses.

## CAMS: bridges across the synaptic cleft

In the brain, neurons recognize each other and form stable synaptic connections through CAMs. CAMs are proteins locate on the cell surface that serve as “glue” for adhering pre- and post-synaptic terminals together. These proteins are responsible for mechanical stabilization of and organization of synaptic contacts. Typically, they contain three domains: an intracellular domain that interacts with the intracellular scaffold protein, a transmembrane domain, and an extracellular domain that interacts with other CAMs via either *trans*- or *cis*-interactions. Most CAMs (e.g., neurexins and neuroligins, SynCAMs, and β1 integrin) localize at the center of the synapse (Mortillo et al., 2012); whereas others (e.g., the N-cadherin/β-catenin system) are found at the outer rims of pre-synaptic active zones and post-synaptic regions (Uchida et al., 1996).

The CAMs play a crucial role in determining synapse specificity by mediating the initial target recognition between pre- and post-synaptic neurons during synapse formation (Sanes and Yamagata, 2009; Williams et al., 2010). Synaptic components are enriched at pre- and post-synaptic terminals in the early stages of synapse development with the help of CAM interactions (Dalva et al., 2007; Chavis and Westbrook, 2001). During the later stages of synapse development and in mature synapses, CAMs regulate synaptic structure and function.

### Neurexin and neuroligin

Neurexins (Nrxs) and neuroligins (NLs) form one of the best studied molecule pairs in the CAM family. Nrxs, discovered as receptors for α-latrotoxin (Südhof, 2008), are type-I transmembrane proteins localized on the pre-synaptic membrane (Berninghausen et al., 2007). Three different genes coding for Nrxs (Nrx1, 2, 3) are present in mammalian neurons. Each gene is driven by two different promoters, resulting in two transcripts encoding a long form of α-Nrxs 1–3 and a short form of β-Nrxs 1–3 (Baudouin and Scheiffele, 2010). NLs are also type-I proteins found on the post-synaptic membrane. At present, at least 4 (in mice and rats) or 5 (in humans) NL isoforms have been identified (Lisé and El-Husseini, 2006; Jamain et al., 2008). The Nrxs and NLs both contain an extracellular domain that participates in pre- and post-synaptic interactions and an intracellular domain that is involved in multiple functional interactions and regulation processes (Südhof, 2008; Lisé and El-Husseini, 2006; Craig and Kang, 2007).

Nrxs and NLs interact with each other with high affinity via their extracellular regions (Scheiffele et al., 2000; Comoletti et al., 2006). The crystal structures of Nrxs and NLs indicate that these extracellular parts form a trans-synaptic complex in the synaptic cleft (Araç et al., 2007). The binding of Nrxs and NLs is Ca^2+^-dependent (Boucard et al., 2005; Chen et al., 2008; Ichtchenko et al., 1995), as confirmed by experiments showing Ca^2+^-dependent cell-cell adhesion following the mixing of two cell populations separately expressing NL-1 and β-Nrx (Boucard et al., 2005; Nguyen and Südhof, 1997). Five canonical alternative splice sites are identified in α-Nrxs and two in β-Nrxs (Resnick et al., 2008; Tabuchi and Südhof, 2002), which predict more than a thousand different splicing transcripts for Nrxs (Ullrich et al., 1995; Missler et al., 1998). As shown by electron studies, spliced sequence #2 (SS2) and #3 (SS#3) are located in the core structure of extracellular domain of Nrxs, while SS#1 and SS#5 are found in distorted regions (Chen et al., 2011). Different from other splicing sites, SS#4, which locates between the fifth and sixth laminin-nectin-sex-hormone binding globulin (LNS) domain, displays the most significance to affect the binding affinity to NLs and other proteins among the splicing sites (Boucard et al., 2005; Chih et al., 2006; Reissner et al., 2013). NLs have two alternative splicing sites (splice site A and B) (Ichtchenko et al., 1995). Different splicing variants display distinct frequency of occurrence and region specific expression, indicating their synapse- or cell- specific roles (Ullrich et al., 1995). The binding affinities of Nrxs and NLs are controlled by alternative splicing of both molecules (Comoletti et al., 2006; Boucard et al., 2005; Chih et al., 2006). The α-Nrxs and β-Nrxs both bind to NL1 that lacks splice site B, and are independent of SS#4 in Nrxs. In the presence of splice site B, NL1 binds only to β-Nrxs, but does not bind to α-Nrxs, without SS#4 (Boucard et al., 2005). This apparent splice insert dependency of Nrx/NL interaction raises a splice-code hypothesis that specific pairings of Nrx/NL complex according to their roles at different location (Nam and Chen, 2005; Boucard et al., 2005; Ichtchenko et al., 1995; Chih et al., 2006).

The C-terminal of Nrxs and NLs interact with intracellular scaffolding proteins to mediate pre- and post-synaptic differentiation and function. Nrxs bind CASK (Ca^2+^/calmodulin-activated Ser-Thr kinase) in the pre-synaptic terminal, while CASK binds Velis/MALs proteins and Mints/X11 proteins (Butz et al., 1998; Borg et al., 1999). In addition, CASK phosphorylates the c-tail of Nrxs in an activity-dependent manner (Mukherjee et al., 2008), suggesting that Nrxs link extracellular protein-protein interactions with intracellular signaling cascade. NLs bind to PSD-95 (post-synaptic density-95), which is the core scaffolding protein at glutamatergic synapses. At post-synaptic sites, the NLs/Nrxs interaction causes an increase in PSD-95 clustering and the recruitment of post-synaptic NMDA (N-methyl-D-aspartate) and AMPA (α-amino-3-hydroxy-5-methyl-4-isoxazolepropionic acid) receptors (Nam and Chen, 2005; Heine et al., 2008; Chih et al., 2005; Barrow et al., 2009). Thus, the binding of Nrxs and NLs to their partners, helps to align the pre-synaptic release machinery and post-synaptic receptors.

How exactly the Nrx/NL complex functions at synapses? Initial studies showed that the expression of NLs in non-neuronal cells induces pre-synaptic differentiation at the contacting axons of cultured neurons, whereas expressing β-Nrx in non-neuronal cells induces post-synaptic differentiation at the contacting dendrites from neurons. The synaptogenic effects of Nrxs are dependent on the LNS domain (Gokce and Südhof, 2013). Overexpression of NLs in cultured neurons increases synapse numbers in a synapse-type and NL-isoform-dependent manner (Chih et al., 2004). For example, overexpression of NL2 in neurons specifically increases the number of inhibitory synapses, but not the excitatory synapses, which is consistent with the preferred localization of NL2 at inhibitory synapses (Chih et al., 2005; Varoqueaux et al., 2004; Levinson et al., 2005). Suppression of NL expression by RNA interference (RNAi) or disruption of Nrx/NL interaction consistently reduces the number of synapses (Chih et al., 2005; Levinson et al., 2005). Thus, *in vitro* studies suggest that Nrx/NL interactions promote synapse formation and may be necessary for synapse stability.

*In vivo* analysis from knockout (KO) mice showed that NLs and Nrxs are essential for synaptic maturation and function (Varoqueaux et al., 2006; Missler et al., 2003; Chubykin et al., 2007). The α-Nrx KO mice show significant impairments in Ca^2+^-triggered neurotransmitter release at both excitatory and inhibitory synapses, possibly due to effects on the pre-synaptic organization of voltage-gated Ca^2+^ channels (Missler et al., 2003). KO of NL1 in mice reduces the synaptic strength at excitatory synapses, whereas the neurons lacking NL2 show synaptic dysfunction at inhibitory synapses. NL1–3 triple KO mice are neonatal lethal, and massive synaptic impairments have been observed from both *in vitro* and *in situ* analysis of these mice. KO of NL1–3 in neurons has no effect on the density of synapses in either the brain or in cultured neurons. However, the expression levels of many synaptic proteins, and the basal synaptic transmission and neural network activity are severely impaired (Varoqueaux et al., 2006). These data suggest that Nrxs and NLs are important in maintaining the basal synaptic transmission. In addition, Nrxs and NLs also contribute to the long-term plasticity of synapses via an activity-dependent mechanism (Varoqueaux et al., 2004). The hippocampal dentate gyrus shows inhibition of long-term potentiation (LTP) in NL1-Null mice (Jedlicka et al., 2013). Constitutive inclusion of an alternatively SS4 in Nrx-3 impairs the recruitment of the post-synaptic AMPA receptor (AMPAR) in mice during NMDA receptor (NMDAR)-dependent LTP (Aoto et al., 2013).

The results showing NL1 and NL2 act on excitatory and inhibitory synapses, respectively suggests an attractive hypothesis; namely, that the excitation/inhibition ratio could be regulated by relative expression levels of NL1 and NL2. Indeed, the amounts of NL1 and NL2 in glutamatergic and GABAergic synapses are restricted by small extracellular splice insertions. The GABAergic associated NL isoforms bind to α-Nrx1 and a subset of β-Nrx1, resulting in GABAergic but not glutamatergic post-synaptic differentiation (Chih et al., 2006). Together, Nrx/NL interactions are sufficient but not absolutely required for synapse formation, as revealed by other KO studies. Other CAM proteins may therefore contribute redundant intercellular functions.

### LRRTMs

The LRRTM proteins are a group of brain-enriched type-I transmembrane proteins that contain extracellular leucine-rich repeats and a short cytoplasmic tail. Four known LRRTMs are recognized (LRRTM 1–4) and are mainly located at excitatory synapses. The LRRTM family is expressed in both developing and adult brains and is especially enriched in the post-synaptic density (PSD) (Laurén et al., 2003). Non-neuronal cells expressing LRRTMs induce pre-synaptic differentiation when co-cultured with hippocampal neurons (Linhoff et al., 2009). *In vitro* assays identify that knocking down LRRTM2 reduces, whereas overexpression of LRRTM2 increases, the number of excitatory synapses, but not inhibitory synapses (de Wit et al., 2009; Ko et al., 2009). The extracellular LRR domain of LRRTM2 is considered to induce this excitatory pre-synaptic differentiation (Siddiqui et al., 2013). LRRTM4-Null dentate gyrus granule cells show reduced numbers of excitatory synapses and impairments in both miniature and action-potential-evoked synaptic transmission at excitatory synapses (Siddiqui et al., 2013). Recently, both α- and β-Nrxs were identified as LRRTM2 ligands. Although the LRRTM-Nrx interaction plays a key role in regulating excitatory synapse formation, the binding of LRRTMs to Nrxs has a distinct regulatory mechanism that involves NLs. LRRTM2 only binds to Nrxs that lack an insert in SS#4 whereas NLs bind to Nrxs regardless of the presence or absence of an insert in SS#4. Recombinant β-Nrx1 also blocks LRRTMs/Nrxs binding (Ko et al., 2009).

Since LRRTMs and NLs can both bind to Nrxs, an interesting question is raised regarding whether LRRTMs and NLs are functionally redundant, cooperative, or antagonistic. Single, double, or triple knockdowns of LRRTM1, LRRTM2, and NL-3 in cultured hippocampal neurons have no effect on synapse numbers, whereas triple knockdown (TKD) of two LRRTMs and NL-3 in cultured NL-1 KO neurons leads to a ~40 % reduction in excitatory synapses (Ko et al., 2011). Knockdown of LRRTM1 and LRRTM2 selectively reduces AMPA receptor-mediated synaptic currents, while knockdown of both LRRTMs, together with NL-3, reduce AMPAR and NMDAR-mediated currents in NL-1 deficiency mice in synapses forming stage (Soler-Llavina et al., 2011). Knockdown of NL-3 at the early stages of synapse formation has no effect on excitatory synaptic transmission regardless of NL-1 expression. These data clearly suggest a functional redundancy between NLs and LRRTMs in developing excitatory synapses. However, LRRTMs and NLs may act differently in mature synapses. For example, inactivation of LRRTM expression, starting from P21 to P35–40, has no effect on excitatory synaptic transmission, while knockout of NL1 reduces the NMDAR/AMPAR ratio at similar ages (Soler-Llavina et al., 2011). In addition, mice lacking LRRTM1 exhibit an increase in the size of pre-synaptic terminals in the hippocampal CA1 region, and an extraordinary phenotype where the animals show avoidance of small enclosures, an increase in social interaction, and a decrease in nest building (Linhoff et al., 2009; Voikar et al., 2013). In acute hippocampal slices, double knockdown of LRRTM1 and LRRTM2 impairs LTP, which can be rescued by the expression of the LRRTM2 extracellular domain (Soler-Llavina et al., 2013). These results indicate that LRRTMs not only play a key role in synapse development and maturation, but also are directly involved in synaptic transmission and more complicated behaviors.

### N-cadherin/β-catenin

Cadherins, a large superfamily of CAMs (more than 100 members in humans), are grouped into subfamilies of classic cadherins and protocadherins. These are also transmembrane proteins containing an extracellular domain with a repeated “cadherin motif” or “cadherin repeat” sequence (Takeichi, 1988).

N-cadherin is the most abundant cadherin in excitatory synapses in the brain. It has five extracellular cadherin motifs and a highly conserved cytoplasmic domain that can bind β-catenin and p120-catenin (Takeichi, 1988; Takeichi, 2007). N-cadherin mediates Ca^2+^-dependent homophilic protein interactions (Hirano and Takeichi, 2012). During synaptic maturation, the location of N-cadherin shifts from the cleft of the synapses to the outer rims of the active zone (Uchida et al., 1996; Fannon and Colman, 1996). Synapse maturation in the active zone is associated with the clustering of N-cadherin at puncta adherentia junctions (PAJs) (Benson and Tanaka, 1998; Tallafuss et al., 2010).

Classical cadherins bind to β-catenin at its central armadillo repeat domain, and β-catenin interacts with the actin cytoskeleton through α-catenin. Functional studies reveal important synaptic functions of cadherins and β-catenin, especially at the excitatory synapses. Cultured neurons lacking N-cadherin or β-catenin show impairments in the development of post-synaptic spines, including reduced spine number, more filopodia-like spines, thinner spines, or spines with smaller heads (Mendez et al., 2010; Saglietti et al., 2007; Okuda et al., 2007). Hippocampal conditional KO mice show reductions in the stability of coordinated spine enlargement and LTP in the CA1 region, with spine density, morphology, and basal synaptic neurotransmission untouched (Bozdagi et al., 2010). The LTP-induced long-term stabilization of synapses is also impaired in expression mutants or knockdown of N-cadherin (Mendez et al., 2010). The cooperation between NL1 and N-cadherin has recently been revealed to promote the formation of glutamatergic synapses in hippocampal cultures and control vesicle clustering at nascent synapses (Aiga et al., 2011; Stan et al., 2010). N-cadherin is also thought to interact with the AMPA receptor subunit GluA2, thereby regulating the expression and trafficking of AMPARs (Saglietti et al., 2007; Nuriya and Huganir, 2006). These results suggest that N-cadherin-mediating adhesion may be responsible for dendritic spine stabilization and synaptic transmission. In primary hippocampal cultures, the suppression of β-catenin expression decreases the amplitude but not the frequency of spontaneous excitatory synaptic currents. Similar treatment impairs synaptic scaling induced by a two-day blockade of neural activity with tetrodotoxin or bicuculline (Okuda et al., 2007). Down regulation of acetylcholine receptor (AChR) clustering by β-catenin also results in an inhibition of post-synaptic differentiation at the neuromuscular junction (Wang and Luo, 2008).

Beyond the post-synaptic functions, N-cadherin and β-catenin are also involved in regulating pre-synaptic vesicle exocytosis. Overexpression of the extracellular domain of N-cadherin increases the frequency of miniature excitatory post-synaptic currents (mEPSCs) (Saglietti et al., 2007). The absence of N-cadherin dramatically impairs short-term plasticity from facilitation to depression at glutamatergic synapses (Jüngling et al., 2006). Mice deficient in β-catenin show a reduction in the number of reserved pool vesicles and impairment in their response to prolonged repetitive stimulation (Bamji et al., 2003). Recently, axonal knockdown of β-catenin has been shown to affect the dynamics of vesicle release (Taylor et al., 2013). Therefore, N-cadherin and β-catenin are structurally and functionally linked in the processes of synapse stabilization as well as in the processes of synaptic transmission from both sides of the synapses. Since the N-cadherin/β-catenin complex stands at the intersection between pre- and post-synaptic functions, it is important to investigate more for better understanding their functions to connect synaptic sides together.

### Ephrins and Eph receptors

Eph receptors (EphA and B) represent the largest family of receptor tyrosine kinases. Eph receptors contain an extracellular domain that comprises a globular ephrin ligand-binding domain, a cysteine-rich region, and two fibronectin type III domains; a cytoplasmic domain composed of a juxtamembrane region with two conserved tyrosine residues; a tyrosine kinase domain; a sterile alpha motif (SAM); and a PDZ-binding motif (Kullander and Klein, 2002; Himanen, 2012). EphA receptors bind to glycosylphosphatidylinositol (GPI)-anchored proteins ephrinA, while EphB receptors bind to transmembrane ephrinB ligands. EphA4, a known exception, can bind to both classes of ephrins. Eph and ephrin expressions are not restricted to synapses: they are found at both the pre- and post-synaptic membranes, and also, at least some isoforms, on astrocytes (Klein, 2009). Eph signaling functions in both developing and mature synapses. Eph-ephrin interaction also could mediate signal transductions between the receptor-expressing cells and the ligand-expressing cells in a bidirectional manner (Daar, 2012).

Eph and ephrin signaling is involved in many regulation processes, including axon guidance and cell migration (Davy and Soriano, 2005; Xu and Henkemeyer, 2012; Egea and Klein, 2007). The activation of cyclin-dependent kinase 5 (Cdk5) and ephexin1 by ephrin-A1 promotes EphA4-dependent spine retraction, followed by a scaling-down of excitatory synaptic strength (Fu et al., 2007; Peng et al., 2013). EphA4 also inhibits integrin signaling pathways (Bourgin et al., 2007), and EphA4 activation by ephrin-A3 reduces tyrosine phosphorylation of the scaffolding protein Crk-associated substrate (Cas), the tyrosine kinase focal adhesion kinase (FAK), and proline-rich tyrosine kinase 2 (Pyk2) while down-regulating the association of Cas with the Src family kinase Fyn and the adaptor Crk. The EphA4 receptor linked with spine-associated RapGAP (SPAR), which is activated by GTPase, regulates the activities of the Rap GTPase, and therefore neuronal morphology (Richter et al., 2007). Cortical neurons with enhanced expression levels of EphA4 show increased numbers of mature spines (Clifford et al., 2011). EphA4 KO mice are disorganized, confirming an involvement of EphA4 forward signaling in the process of dendritic spine maturation (Murai et al., 2003). Remodeling of the spines by the EphA receptor rearranges the distribution of F-actin in spines (Zhou et al., 2012).

EphA-ephrinA signaling shapes the synaptic strength in addition to regulating cell morphology (Hruska and Dalva, 2012). The activation of EphA4 decreases synaptic and surface GluR1 and attenuates mEPSCs amplitude through an APC (Cdh1)-dependent degradation pathway (Fu et al., 2011). The Eph4-deficient hippocampal CA1 region shows impairment of LTP and long-term depression (LTD); this impairment is independent of the cytoplasmic domain of Eph4, suggesting that ephrinBs are the active signaling partners (Grunwald et al., 2004). Amygdala neurons also have a requirement for EphA4 for synaptic plasticity. Rin1, a brain-specific Rab5-GEF, mediates EphA4 endocytosis and down-regulates EphA4 signaling, which in turn affects LTP (Deininger et al., 2008). Post-synaptic expression of EphA4 and its ligand ephrin-A3 in astrocytes mediates neuron-glia interactions, which are also required for LTP expression at CA3-CA1 synapses in the hippocampus (Filosa et al., 2009).

EphB-ephrinB signaling at synapses is also well studied. EphrinB3 expression is related to glutamatergic synapse density on the dendritic shafts, but not on the spines (Aoto et al., 2007). EphrinB binding to the EphB receptor elevates excitatory synapse formation via degradation of Ephexin5, a RhoA guanine nucleotide exchange factor (Margolis et al., 2010). Suppression of the expression of the EphB receptor reduces excitatory glutamatergic synapses and the clustering of NMDARs and AMPARs, and alters dendritic spine formation as well (Henkemeyer et al., 2003). The PDZ domain of EphB2 also controls localization of the AMPA-type glutamate receptor, while the ephrin binding domain of EphB2 initiates pre-synaptic differentiation (Kayser et al., 2006). EphBs are thought to control synaptogenesis by associating the motility of filopodia and the binding ability of ephrin (Kayser et al., 2008). The Rho-GEF kalirin, Rac1, and its effector PAK are involved in the ephrinB-EphB signaling pathway during spine development (Penzes et al., 2003). Tiam1, a Rac1 guanine nucleotide exchange factor, is phosphorylated by EphBs and promotes Rac1-dependent actin cytoskeletal remodeling for dendritic spine morphogenesis (Tolias et al., 2007). Together, these data indicate that EphB-ephrinB signaling promotes excitatory synaptogenesis.

In addition to its synaptogenesis function, EphB-ephrinB signaling also plays an important role in regulating synaptic plasticity. The suppression of EphB2 expression by siRNA in the post-synaptic neuron reduces mEPSCs frequency in cultured cortical neurons (Kayser et al., 2006). EphB2 deficient mice show reduced NMDA-mediated synaptic responses and impaired LTP (Henderson et al., 2001). This impairment of LTP can be rescued by expressing C-terminal truncated EphB2, indicating that EphB2 kinase signaling is not responsible for these functions (Grunwald et al., 2001). The tyrosine phosphorylation sites in ephrinB2 are necessary for maintaining LTP but not LTD, whereas the C-terminal PDZ interaction site is required for both (Bouzioukh et al., 2007). EphrinB3-deficient mice show reduced amplitude of mEPSCs, but increased NMDA/AMPA ratios in CA1 neurons (Antion et al., 2010). Blocking the interaction between EphRs and the PDZ protein GRIP or extracellular application of soluble forms of B-ephrins (which are pre-synaptic ligands for the EphB receptors) reduces mossy fiber LTPs in the CA3 region, suggesting a requirement for trans-synaptic interactions between post-synaptic EphB receptors and pre-synaptic B-ephrins (Contractor et al., 2002). Replacement of the cytoplasmic C-terminal signaling domain of the ephrinB3 with β-galactosidase selectively blocks mossy fiber LTPs (Armstrong et al., 2006). Therefore, trans-synaptic ephrin-Eph adhesion regulates synaptic maturation and plasticity in a bidirectional way in both developing and adult brains.

### NCAM

The neural cell adhesion molecule (NCAM) is a glycoprotein of the immunoglobulin (Ig) superfamily, expressed in both the pre- and post-synaptic membranes. The extracellular part of NCAM has five Ig domains that bind to NCAM, and two fibronectin type III (FNIII) domains related to neurite outgrowth. At least 27 alternatively spliced NCAM mRNAs are present in rat brain, suggesting wide and diverse functions of NCAM (Reyes et al., 1991).

Numerous studies have shown that NCAM regulates synapse formation, maturation, and function through homo- and hetero-philic interactions (Bukalo and Dityatev, 2012). The ablation of NCAM reduces the number of synapses (Dityatev et al., 2000). NCAM controls axonal branching and button formation in GABAergic synapses in basket interneurons (Chattopadhyaya et al., 2013). NCAM associates with the post-synaptic spectrin-based scaffold to form a complex that is responsible for recruiting NMDARs and Ca^2+^/calmodulin-dependent protein kinase II alpha (CaMKIIalpha) to synapses and is important for NMDAR-dependent LTP and LTD (Sytnyk et al., 2006; Bukalo et al., 2004; Muller et al., 1996). Therefore, NCAM recruits the NMDAR and other PSD components for both synapse formation and synaptic plasticity.

NCAM also has pre-synaptic functions. Deleting NCAM at the neuromuscular junction (NMJ) leads to smaller NMJs and impaired accumulation of pre-synaptic proteins. The number of docked vesicles is reduced and the paired-pulse facilitation (PPF) is lacking at NCAM null junctions (Rafuse et al., 2000). Multiple alterations including vesicle mobilization/cycling in pre-synaptic terminals are also observed in NCAM-deficient mice (Polo-Parada et al., 2001). The C-terminal of NCAM plays a key role in maintaining effective transmission via a pathway involving myosin light chain kinase (MLCK) and probably MLC and myosin II (Polo-Parada et al., 2005). This pathway is thought to control the replenishment of synaptic vesicles during high levels of exocytosis through the facilitation of myosin-driven delivery of vesicles to active zones for subsequent exocytosis. Chromaffin cells show impairment of catecholamine granule trafficking between the readily releasable pool and the highly release-competent immediately releasable pool in the absence of NCAM, resulting in a reduced rate of granule fusion under physiological stimulation. These findings suggest that NCAM is involved in vesicle recycling in both neuronal and endocrine cells (Chan et al., 2005).

### L1-CAMs

The L1 is a family of transmembrane proteins, known as neuronal cell adhesion molecules (L1-CAMs). At least four members are recognized in vertebrates: L1CAM, Close Homolog of L1 (CHL1), NgCAM-related cell adhesion molecule (NrCAM), and Neurofascin. L1CAM contains an ectodomain with six Ig-like domains and five fibronectin type III repeats, followed by a transmembrane region and a highly conserved cytoplasmic tail (Moos et al., 1988). The intracellular domain of L1 interacts with many other synaptic organizers, including ankyrin, actin, spectrin, and 14-3-3 proteins (Hortsch et al., 2009; Ramser et al., 2010; Herron et al., 2009; Loers and Schachner, 2007). The L1-CAMs are involved in many neuronal functions, including axonal guidance, neurite outgrowth and fasciculation, and cell migration (Chang et al., 1987; Lindner et al., 1983; Fischer et al., 1986; Maness and Schachner, 2007).

L1-deficient mice show a significant reduction in frequency, but not amplitude, of miniature inhibitory post-synaptic currents (mIPSCs), and a reduction in the mean amplitude of putative unitary IPSCs, whereas the basal excitatory synaptic transmission is normal (Saghatelyan et al., 2004). However, the conditional inactivation of L1 in the adult brain increases the basal excitatory synaptic transmission and decreases anxiety in the open field, which differs from the response seen in L1 constitutive KO mice (Law et al., 2003). These differences might arise from the developmental function of L1, as no structural abnormalities in morphology are observed in these mice when compared to constitutive KO mice. The L1/ankyrin interactions are important in regulating the functions of inhibitory synapses. The ankyrin-mediated localization of L1CAMs is implicated in the organization of GABAergic synapses in Purkinje neurons (Ango et al., 2004). Loss of the L1/ankyrin interaction impairs branching of GABAergic interneurons and specifically reduces the number of perisomatic synapses (Guan and Maness, 2010).

CHL1, another member of the L1 subfamily, has a reported involvement in synaptogenesis of inhibitory interneurons, although it functions differently from L1. The hippocampal CA1 region in juvenile CHL1 mutant mice shows an increase in inhibitory post-synaptic currents and a decrease in LTP at CA3-CA1 excitatory synapses. The length and linear density of active zones, and the numbers of perisomatic puncta containing inhibitory axonal markers, are also increased (Nikonenko et al., 2006). CHL1-deficient mice show enhancement of basal synaptic transmission in the lateral and medial perforant path projections to the dentate gyrus, whereas reactivity to environmental stimuli and expression of social behaviors are reduced (Morellini et al., 2007). CHL1 also maintains inhibitory synapses between stellate axons and Purkinje dendrites, indicating a role in connecting glia and neuron (Ango et al., 2008).

IgCAMs, including the previously mentioned NCAM and L1CAM, are capable of binding in both *trans-* and *cis-* orientations. Early structural studies identified the involvement of the multiple Ig domains of NCAM and L1 in *trans* binding (Bateman et al., 1996; De Angelis et al., 1999; Jensen et al., 1999). Both NCAM and L1 family members could be palmitoylated and targeted to lipid rafts, indicating *cis* interactions between these CAMs (Little et al., 1998; Ren and Bennett, 1998). NCAM and L1 can stimulate neurite growth via a mitogen-activated protein kinase (MAPK) dependent pathway. In PC12 cells, the MAP kinase extracellular signal-regulated kinases ERK1 and ERK2 are phosphorylated through interaction of NCAM with a synthetic NCAM peptide ligand (Kolkova et al., 2000). In 3T3 cells, L1 cross-linking can activate ERK2, a component of the MAPK cascade (Schaefer et al., 1999). The fibroblast growth factor (FGF) receptor is also proposed to share downstream signaling pathways with CAMs during the stimulation of neurite growth (Kolkova et al., 2000).

These CAMs transduce signals from outside to inside, but they also can transduce signals in the opposite direction because of their functions in cell migration and synaptic plasticity. For example, L1 can bind ankyrin, resulting in oligomerization of CAMs and an enhancement of homophilic trans-adhesion on the membrane (Tuvia et al., 1997). Different members of L1CAMs can interact with ankyrin to form hetero-oligomers with different affinities, and different regulation by homophilic or heterophilic ligand binding to the CAMs (Malhotra et al., 1998). Together, the CAMs mediate the adhesion response to external stimuli, and inside-out signaling transduction.

### Nectins

Nectins are Ca^2+^-independent Ig-CAMs (Takai et al., 2003). At present, four nectins have been identified in humans. All nectins can form homo-*cis* dimers followed by *trans*-interaction in an either heterophilic or homophilic manner through their extracellular domains (Mizoguchi et al., 2002). Nectin-3 interacts with Nectin-1 or -2 to form a hetero-trans-dimer with a higher binding ability than homo-trans-dimers (Rikitake et al., 2012).

Nectins interact with actin-binding protein afadin, an α-catenin interacting protein, through the C-terminal PDZ binding domain, which predicts an involvement with the cadherin/catenin adhesion complex (Giagtzoglou et al., 2009). Nectin-1 and afadin form clusters at developing synapses, and these clusters colocalize with the N-cadherin-catenin complex, implying a role of these two bimolecular pairs during initial synapse formation. Similar to N-cadherin, Nectin-1 mainly locates at matured excitatory synapses although it is initially found at both excitatory and inhibitory synapses (Lim et al., 2008). The CA3 area of the adult hippocampus shows an asymmetric localization of Nectin-1 and -3 at the pre- and post-synaptic sides, in contrast to the symmetrically localization of afadin expression. Reduction in nectin-based adhesion leads to a decrease in synapse size and an accompanying increase in synapse number, suggesting a role of the nectin-afadin system in synaptogenesis (Mizoguchi et al., 2002). Mice deficient in either Nectin-1 or Nectin-3 show a reduced number of puncta adherentia junctions (PAJs) and abnormal mossy fiber trajectory (Honda et al., 2006). Nectin-1, but not Nectin-3, plays a role in increasing contextual fear memory (Fantin et al., 2013). On the other hand, conditional absence of afadin largely reduces the signal of nectins, N-cadherin, and β-catenin, and disrupts PAJs, whereas it increases the numbers of perforated synapses. Thus the nectin-afadin interaction appears to participate in synaptic remodeling by regulating the stability of synaptic junctions (Majima et al., 2009).

The nectin-afadin complex also interacts with many synaptic proteins that function at synapses. The synaptic scaffolding molecule (S-SCAM) has been reported to co-localize with nectins via the PDZ domain-binding domain of the latter (Yamada et al., 2003). S-SCAM is involved in the pre-synaptic vesicle clustering mediated by N-cadherin and NL-1 cooperation (Stan et al., 2010). NL-1 induces the release probability and enhances mEPSCs frequency in the presence of N-cadherin. Several cell adhesion molecules therefore can function either separately or synergistically in synapse maturation (Sakisaka et al., 2007).

### Contactins

Contactins (CNTN) are a group of GPI-linked Ig-CAMs containing six N-terminal Ig-like domains and four fibronectin III-like domains. Six members are recognized in the CNTN family: CNTN-1, CNTN-2/TAG-1, CNTN-3/BIG-1, CNTN-4/BIG-2, CNTN-5/NB2, and CNTN-6/NB3. CNTNs play an important role in the formation of axon connections in the developing nervous system. Both CNTN-1 and CNTN-2 are involved in axon growth and guidance (Buttiglione et al., 1996; Perrin et al., 2001). CNTN-6 is prominently expressed pre-synaptically in the developing nervous system. Hippocampal neurons show co-expression of CNTN-6 with the excitatory synaptic markers vesicular glutamate transporter 1 (VGLUT1) and 2 (VGLUT2), but not with the inhibitory synapse marker vesicular GABA transporter (VGAT). CNTN-6 deficient mice show increased numbers of immature granule cells in the internal granule cell layer (IGL) and a decreased density of parallel fiber synaptic terminals in the cerebellum (Sakurai et al., 2009). KO of CNTN-6 selectively reduces excitatory but not inhibitory synapse density (Sakurai et al., 2010). Thus, CNTN-6 seems to be required for postnatal glutamatergic synapse development. CNTN 4 and 5 are also involved in synapse differentiation, especially at early stages. CNTN-4 extends the length of neurites, while CNTN-5 increases the number of roots (Mercati et al., 2013). Unlike the pre-synaptic localization of CNTN-6, CNTN-1 has been detected in PSD in CA1 pyramidal cells. Inactivation of CNTN-1 expression leaves the basal transmission and LTP level intact, without altering synaptic morphology either (Murai et al., 2002), but PPF and NMDA receptor-dependent LTD are impaired in these mice. In adult mice, overexpression of contactin increases LTP and spatial and object recognition memory (Puzzo et al., 2013). CNTNs may therefore function at both pre- and post-synaptic sites, although the mechanisms remain unclear.

### SynCAM

Synaptic cell adhesion molecules (SynCAM) have been identified as a family of proteins that contain three extracellular Ig-like domains, a single transmembrane domain and a cytoplasmic tail (Thomas et al., 2008). Four SynCAM isoforms are recognized: SynCAMs 1–4. All SynCAMs are highly enriched in the brain, and SynCAM 1 is also found in the lung and testis (Fogel et al., 2007). Like the NLs, SynCAM recruits synaptic proteins and promotes neuron differentiation pre-synaptically in co-culture assays (Sara et al., 2005). During synapse development, SynCAMs are located in both pre- and post-synaptic plasma membranes and undergo homo- and hetero-philic adhesive interactions. Interestingly, unlike NCAM and L1CAM, SynCAMs are preferentially assembled into heterophilic rather than homophilic complexes. SynCAMs 1 and 2 bind to each other across the synaptic cleft to form a trans-synaptic SynCAM 1/2 complex that is subject to glycosylation modifications (Fogel et al., 2007). The number of pre-synaptic terminals and the level of excitatory synaptic transmission are increased via the binding of SynCAM 1 and 2 (Fogel et al., 2007; Sara et al., 2005). Consistently, loss of SynCAM 1 decreases excitatory synapse number in the nucleus accumbens (Giza et al., 2013). Lateral self-assembly of SynCAM 1 has also been reported. This lateral interaction is required for synaptogenic activity in immature neurons, while restricting synaptic size in mature synapses (Fogel et al., 2011).

In addition to their homo- and hetero-philic interaction, SynCAMs also bind many other proteins via the C-terminal domain. SynCAMs bind to the scaffold proteins syntenin and CASK via the C-terminal PDZ domain, and recruit CASK to the plasma membrane (Biederer et al., 2002). SynCAM1 also binds to protein 4.1B intracellularly, which in turn recruits NMDAR to the post-synaptic plasma membrane, resulting in an increase in the frequency of NMDAR-mediated mEPSCs in cultured hippocampal neurons (Hoy et al., 2009).

*In vivo* studies have revealed a role for SynCAM 1 in the regulation of synapse numbers and plasticity. Mouse neurons form fewer excitatory synapses in the absence of SynCAM 1, while overexpression of SynCAM 1 results in an increase in excitatory synapse number. The LTD and spatial learning are also regulated by the expression of SynCAM 1 (Robbins et al., 2010).

Although SynCAMs and NLs both function in the pre-synaptic induction of synapses, the mechanisms underlying this response might be distinct. Co-culture assays show that both spontaneous and evoked neurotransmitter release induced by SynCAM and NLs are indistinguishable. However, electrophysiological analysis reveals that only SynCAM increases the early development of the excitatory neurons by its intracellular cytoplasmic domain. Morphological analysis shows that only NL1 increases the synapse number and spine density (Sara et al., 2005). These contradictions observed in different assays could be reconciled by assuming that the role of SynCAM is to increase the vesicle pool size of previously existing synapses whereas the role of NL1 is to lead synapse formation without altered recruitment of AMPARs in the post-synaptic site or proper assembly of pre-synaptic secretory apparatus. Therefore, proper synaptogenesis probably requires more than just one ‘master molecule’, with many molecules functioning instead, either separately or in cooperation, in the discrete steps of synapse formation.

### SALMs

Synaptic adhesion-like molecules (SALMs) are a newly discovered family of adhesion molecules: at least five members have been identified in the central nervous system. SALMs 1–3 contain an extracellular region consisting of a leucine-rich repeat (LRR), a fibronectin type III domain, Ig-like domains, a transmembrane domain, and a C-terminal PDZ-binding motif that interacts with PSD-95. SALMs 4 and 5 lack the PDZ-binding domain (Seabold et al., 2008). SALMs 1–3 bind to each other, while SALMs 4 and 5 form homomeric complexes in brain. Transfected heterologous cells show that only SALMs 4 and 5 form homomeric associations mediated by the extracellular N-terminus (Seabold et al., 2008).

SALMs undergo multiple interactions with other proteins. SALM1 interacts with post-synaptic NMDA receptors, possibly through the extracellular or transmembrane regions, and with scaffold proteins PSD-95, SAP 97, and SAP 102 via the PDZ-binding domain. Immunostaining experiments show that SALM1 recruits PSD-95 and NMDA receptors to post-synaptic sites (Wang et al., 2006; Seabold et al., 2012). SALM2 interacts with PSD-95 and other post-synaptic proteins, including guanylate kinase-associated protein (GKAP) and AMPA receptors at excitatory synapses (Ko et al., 2006). SALM3 and SALM5 recruit VGluT and VGAT (which are pre-synaptic proteins localized at excitatory and inhibitory synapses, respectively), the pre-synaptic vesicle protein synaptophysin, and the pre-synaptic active zone protein Piccolo, although SALM3 has higher affinity for complex formation with PSD-95 compared to SALM5 (Mah et al., 2010). Functional assays revealed that overexpression of SALMs promotes neurite outgrowth in cultured neurons (Wang et al., 2008). Suppression of SALM2 expression decreases the number of excitatory synapses and dendritic spines, and selectively reduces the frequency but not the amplitude of mEPSCs (Ko et al., 2006). On the other hand, knockdown of SALM5 significantly reduces both spontaneous excitatory and inhibitory synaptic transmissions, affecting both frequency and amplitude (Mah et al., 2010). Thus, SALMs regulate excitatory and inhibitory synapse function through distinct mechanisms.

### NGLs

NGL (netrin-G ligand) proteins are a family of LRR-containing CAMs consisting of three members: NGL1–3. NGLs are mainly located post-synaptically at excitatory synapses. NGL-1 and -2 bind to netrin-G1 and netrin-G2 through their cytosolic tails in an isoform-specific manner (Kim et al., 2006). The LRR domain of NGL-3 interacts with pre-synaptic LAR protein to induce synapse formation (Kwon et al., 2010). PTPσ interacts with NGL-3 to promote a bidirectional synapse formation, whereas PTPδ-NGL-3 interaction induces pre-synaptic differentiation in only a unidirectional manner. Receptor tyrosine phosphatases LAR, the NGL-3 binding partner, is also required for maintaining the number of excitatory synapses and dendritic spines, the expression of surface AMPARs, and the targeting of the cadherin-β-catenin complex (Dunah et al., 2005).

Cultured neurons overexpressing NGL-2 show an increase in the number of dendritic protrusions. Suppression or competitive inhibition of NGL-2 reduced the number of excitatory synapses (Kim et al., 2006). Suppression of NGL-2 or NGL-3 selectively decreases excitatory synaptic currents (Kim et al., 2006; Woo et al., 2009). In the retina, loss of NGL-2 impairs branching of horizontal cell axons that stratify in the outer plexiform layer and reduces synapse formation between horizontal cell axons and rods (Soto et al., 2013). LAR knockdown reduces both the amplitude and frequency of mEPSCs (Dunah et al., 2005). Thus, like other cell adhesion molecules, NGLs are also involved in synaptic function.

### IgLONs

IgLONs are a group of adhesion molecules with three extracellular C2 domains and a GPI anchor attach to the membrane. Four genes are presently identified in this family: LAMP (limbic system-associated membrane protein), OBCAM (opioid-binding cell adhesion molecule), Ntm (neurotrimin), and Kilon. The LAMP, Ntm, and OBCAM molecules interact homophilically with themselves and heterophilically with each other (Lodge et al., 2000; Gil et al., 2002). During development, IgLONs show both overlapping and distinct patterns in protein localization. For example, Kilon is distributed in axons and pre-synaptic terminals at early stages, but is mainly observed in the post-synaptic sites of dendritic and somatic synapses in adults (Miyata et al., 2003; Hashimoto et al., 2008). LAMP alters its location from restriction at post-synaptic sites to wide expression on somata, dendrites, and axons in the process of maturation (Pimenta et al., 1996).

The IgLONs are implicated in synaptogenesis. Overexpression of LAMP or OBCAM increases synapse number in hippocampal neurons (Hashimoto et al., 2009). Consistently, down regulation of OBCAM expression reduces synapse number, impairing synapse formation (Yamada et al., 2007). OBCAM also regulates neuronal activity via a raft-dependent pathway. Overexpression of Kilon reduces synapse number at early stages but increases the number of dendritic synapses in mature neurons with the alteration of lipid raft dependence (Hashimoto et al., 2008). However, more data are needed to determine the precise nature of the IgLON involvement in synaptic transmission and plasticity.

### Integrins

Integrins are transmembrane receptors found in organisms ranging from sponges to mammals. Integrins form heterodimers with two type-I transmembrane chains, α subunit and β subunit. At least eighteen α subunits and eight β subunits are known, resulting in 24 unique heterodimers in mammals. Interaction of integrins with other proteins, including cadherins and Ig-CAMs allow transmission of signals across the plasma membrane in both directions and mediate cell-cell and cell-matrix interactions and communication (Hynes, 2002). Some integrin subunits are concentrated at synapses, indicating a role in synaptic function. For example, post-synaptic β3 integrin directly interacts with AMPARs in primary hippocampal cultures (Cingolani et al., 2008). In addition, β3 integrin binds to the GluA2 subunit of AMPARs through their cytoplasmic tails (Pozo et al., 2012). Through the regulation of AMPAR trafficking, similar to that conducted by N-cadherin, β3 integrin is involved in synaptic scaling. β1 integrin, another integrin subunit, shows an altered expression in either limbic seizures or muscle stretch (Pinkstaff et al., 1998; Chen and Grinnell, 1995). Deletion of post-synaptic β1 integrin increases the expression of N-cadherin and NLs, possibly implying a compensatory effect (Mortillo et al., 2012). Overexpression of β3 integrin in the post-synaptic neurons reduces the amplitude of mEPSCs and alters the subunit composition of AMPAR, while inactivation of β3 integrin abolishes the synaptic scaling induced by pharmacological silencing of neuronal activity (Harburger and Calderwood, 2009). In addition to *cis*-regulation, integrins organize the synapse assembly in a trans-synaptic manner. Deleting β1 integrin only in the pre-synaptic terminals alters the ratio of mature and immature spines numbers in cultured neurons (Ning et al., 2013).

Homeostatic synaptic scaling requires β3 integrin, but the function of this protein in synaptic transmission is still not very clear. Excitatory synaptic currents in primary hippocampal pyramidal neurons are increased or decreased by the overexpression of wild type or dominant-negative β3 integrin, respectively (Cingolani et al., 2008). However, expressing β3 integrin mutants, including wild-type, in constitutively inactive or constitutively active mutants has no differential effects in excitatory synaptic responses (Pozo et al., 2012). Deletion of β3 integrin also leaves LTP, LTD, and short-term plasticity unaltered (McGeachie et al., 2012). More investigations are therefore needed to determine the exact role of integrins in synaptic transmission.

### LAR-RPTPs

Leukocyte antigen-related receptor protein tyrosine phosphatases (LAR-RPTPs) have recently been proposed to have a role in pre-synaptic development. The LAR-RPTP proteins have a single transmembrane domain, two intracellular PTP domains, and an extracellular domain (Pulido et al., 1995). Three vertebrate members (LAR, PTPδ, and PTPσ) and a few invertebrate members have been identified in the family (Chagnon et al., 2004). LAR-RPTPs are widely distributed in the brain. LAR and PTPσ are enriched in glutamatergic synapses, and LAR is associated with AMPARs (Wyszynski et al., 2002; Takahashi et al., 2011); whereas PTPδ mainly localized in inhibitory synapses (Takahashi et al., 2012). LAR-RPTPs regulate synapse formation via various protein interactions; for instance, PTPσ and PTPδ are reported to interact with NGL-3 and to promote synapse formation (Kwon et al., 2010). Overexpression of dominant-negative LAR impairs the normal function of β-catenin-cadherin complex that regulates synaptic differentiation (Brigidi and Bamji, 2011). Similarly, RNAi experiments show a reduction in dendritic targeting of the β-catenin-cadherin complex, suggesting that LAR-RPTPs function in maintaining excitatory synapses and dendritic spines (Dunah et al., 2005). PTPσ and PTPδ are required for excitatory and inhibitory synaptic differentiation, respectively, via interactions with Slit- and Trk-like proteins (Slitrks), a family of proteins belonging to the LRR superfamily (Yim et al., 2013).

Electrophysiology studies have revealed synaptic functions for LAR-RPTPs. Excitatory synaptic transmission is dramatically impaired by overexpression of LAR dominant-negative constructs (Dunah et al., 2005). Similarly, loss of LAR-RPTPs reduces the amplitude and frequency of mEPSCs. Mice deficient in PTPδ show increased PPF and LTP in the hippocampus (Uetani et al., 2000). Surprisingly, receptor protein tyrosine phosphatase σ (RPTPσ) null mice show an increase in PPF and mEPSCs frequency, but reduced LTP (Horn et al., 2012). Activation of LAR-RPTPs results in specific mAChR-LTD, but not mGluR-LTD (Dickinson et al., 2009).

Mice that lack LAR phosphatase domains exhibit spatial learning impairment in performance of the Morris water maze, and are more active in exploration and nest-building (Kolkman et al., 2004). Similar learning impairment has been found in mice lacking PTPδ (Uetani et al., 2000). On the contrary, loss of RPTPσ in mice causes an enhancement in novel object recognition memory (Horn et al., 2012). Several studies suggest important functions of LAR-RPTPs at synapses, but the underlying mechanisms still remain to be established (Table 1).

**Table 1:** Interactions and functions mediated by selected CAMs

Molecules	Synaptic location	Interaction	Function	References
Nrxs	pre	α-latrotoxin, CASK, NLs, LRRTMs, GABA_A_-receptor	α-latrotoxin receptor	Südhof, 2008
			Maintain pre-synaptic differentiation, the basal synaptic transmission and long-term plasticity of synapses	Levinson et al., 2005; Varoqueaux et al., 2004, 2006
			↑ PSD-95, ↑ NMDA, and AMPA receptors	Barrow et al., 2009; Chih et al., 2005; Heine et al., 2008; Nam and chen, 2005
			Relate to ASD, AD, and schizophrenia	Reissner et al. 2013; Sindi et al., 2014; Südhof, 2008
			Induce post-synaptic differentiation (β-Nrx)	Gokce and Südhof, 2013
			Impair the recruitment of the post-synaptic AMPAR (Nrx3)	Aoto et al., 2013
NLs	post	Nrxs, PSD-95	Induce pre- and post-synaptic differentiation and function	Chih et al., 2005; Gokce and Südhof, 2013
			Maintain the numbers of synapses, the basal synaptic transmission and long-term plasticity of synapses	Varoqueaux et al., 2004, 2006
			↑ PSD-95, ↑ NMDA, and AMPA receptors	Barrow et al., 2009; Chih et al., 2005; Heine et al., 2008; Nam and chen, 2005
			Relate to ASD and AD	Sindi et al., 2014; Südhof, 2008
			Maintain LTP and the synaptic strength at excitatory synapses (NL1)	Jedlicka et al. 2013
			↑ The inhibitory synapse numbers (NL2)	Levinson et al., 2005
			↑ Social interactions and communication (NL4)	Jamain et al., 2008
LRRTMs	post	Nrxs	Induce pre-synaptic differentiation (LRRTM1)	Linhoff et al., 2009
			Maintain excitatory synapses numbers and synaptic transmission (LRRTM2 and 4)	de Wit et al., 2009; Ko et al., 2009; Siddiqui et al., 2013
N-cadherin	post	β-catenin, AMPAR subunit GluA2, NL1	Mediates Ca^2+^-dependent homophilic protein interaction	Hirano and Takeichi, 2012
			Regulates the development of post-synaptic spines and dendritic spine stabilization	Mendez et al., 2010; Okuda et al., 2007; Saglietti et al., 2007
			Maintains synaptic transmission, short-term plasticity, LTP, ↑ mEPSCs	Bozdagi et al. 2010; Mendez et al., 2010; Saglietti et al., 2007
			Relates to ASD, AD and BP	Asada-Utsugi et al., 2011; Andreyeva et al., 2012; Chapman et al., 2011; Pagnamenta et al., 2011; Sklar et al., 2008
β-catenin	post	N-cadherin, AChR	Maintains the development of post-synaptic spines and the number of reserved pool vesicles	Bamji et al., 2003; Okuda et al. 2007;
			Induces post-synatic differentiation	Wang and Luo, 2008
			Regulates excitatory synaptic currents	Okuda et al. 2007
			↑ vesicle recycling	Taylor et al., 2013
Eph receptors	both	Ephrin, SPAR	Mediate axon guidance, cell migration, pre-synaptic differentiation, and spine maturation	Clifford et al., 2011; Davy and Soriano, 2005; Egea and Klein, 2007; Murai et al., 2003; Xu and Henkemeyer, 2012
			Mediate neuron-glia interactions (EphA4)	Filosa et al., 2009
			↓ mEPSCs, ↓ LTP and LTD (EphA4)	Deininger et al., 2008; Fu et al., 2011; Grunwald et al., 2004
			Maintain excitatory synapses and the clustering of NMDARs and AMPARs (EphB)	Henkemeyer et al., 2003
			↓ mEPSCs (EphB2)	Kayser et al., 2006;
			Relate to AD	De Strooper, 2003; Simón et al., 2009
Ephrins	both	Cdk5, Eph	Mediate axon guidance, cell migration	Davy and Soriano, 2005; Egea and Klein, 2007; Xu and Henkemeyer, 2012
			Maintain NMDA-mediated current and LTP (ephrinB2)	Bouzioukh et al., 2007; Henderson et al., 2001
			↓ mEPSCs, ↑ NMDA/AMPA ratios, ↓ mossy fiber LTPs (EphrinB3)	Antion et al., 2010; Armstrong et al., 2006; Contractor et al., 2002;
NCAM	both	homo- and hetero-philic interactions	Maintains synapses number	Dityatev et al., 2000
			Controls axonal branching and button formation in GABAerigc synases	Chattopadhyaya et al., 2013
			↑ NMDA receptor, ↑ CaMKIIalphaLTP, LTD	Bukalo et al., 2004; Muller et al., 1996; Sytnyk et al., 2006
			↑ Vesicle recycling	Rafuse et al., 2000; Polo-Parada et al., 2001; Polo-Parada et al., 2005; chan et al., 2005
			Linked with schizophrenia and BP	Conrad and Scheibel, 1987; Vawter et al., 1998a, b
L1-CAMs	pre		Control axonal guidance, neurite outgrowth and fasciculation, and cell migration	Chang et al., 1987; Fischer et al., 1986; Lindner et al., 1983; Maness and Schachner, 2007
			↓ Inhibitory synaptic response (L1)	Saghatelyan et al., 2004
			↑ Vesicle recycling (CHL1)	Ango et al., 2008; Morellini et al., 2007; Nikonenko et al., 2006
Nectins	both	afadin, N-cadherin-catenin complex, S-SCAM	Initial synapses formation	Giagtzoglou et al., 2009; Lim et al., 2008
			Regulate the stability of synaptic junctions	Majima et al., 2009
			↑ Contextual fear memory (Nectin-1)	Fantin et al., 2013
			Relate to AD	Kim et al., 2002; Kim et al., 2011
Contactins	both		Mediate axon connections	Buttiglione et al., 1996; Perrin et al., 2001
			↑ PPF, ↑ LTD, ↑ LTP and spatial and object recognition memory (CNTN-1)	Murai et al., 2002; Puzzo et al., 2013
			Extend the length of neurites (CNTN-4)	Mercati et al, 2013
			↑ root (CNTN-5)	Mercati et al, 2013
			Postnatal glutamatergic synapse development (CNTN-6)	Sakurai et al., 2009, 2010
			Relates to ASD	Cottrell et al., 2011; van Daalen et al., 2011
SynCAM	both	CASK, protein 4.1B and NMDAR, heterophilic interactions	Recruits synaptic proteins and promotes neuron differentiation	Sara et al., 2005
			Post-synaptic scaffolding, NMDAR trafficking, ↑ NMDAR-mediated current, ↓ LTD, not LTP in CA1 (SynCAM-1)	Biederer et al., 2002; Hoy et al., 2009
			↑ Vesicle recycling, ↑ number of pre-synaptic terminals, ↑ excitatory synapse number and synaptic transmission (SynCAM-1 and -2)	Fogel et al., 2007; Robbins et al., 2010; Sara et al., 2005
SALMs	both	PSD-95,SAP 97, SAP 102, NMDAR, GKAP, AMPAR	Post-synaptic scaffolding, NMDAR trafficking (SALM-1)	Seabold et al., 2012; Wang et al., 2006
			↑ Frequency of mEPSCsPost-synaptic scaffolding (SALM-2)	Ko et al., 2006
			Post-synaptic scaffolding, recruit pre-synaptic proteins (SALM-3)	Mah et al., 2010
			↑ Frequency and amplitude of mEPSCsPost-synaptic scaffolding, recruit pre-synaptic proteins (SALM-5)	Mah et al., 2010
NGLs	post	netrin-G, LAR, PTPσ, PTPδ	Post-synaptic scaffolding	Dunah et al., 2005; Kwon et al., 2010
			Maintains excitatory synapses and synaptic currentsInduces branching of horizontal cell axons (NGL-2)	Kim et al., 2006; Soto et al., 2013
			Maintain excitatory synaptic currents (NGL-3)	Woo et al., 2009
IgLONs	both	homo- and hetero-philic interactions	Post-synaptic scaffolding	Hashimoto et al., 2008
			↑ Synapse number (LAMP, OBCAM)	Hashimoto et al., 2009
			↓ Synapse number at early stages, ↑increases number of dendritic synapses in mature neurons (Kilon)	Hashimoto et al., 2008
Integrins	both	cadherins, AMPAR	Maintains ratio of mature and immature spines numbers (Integrin-β1)	Ning et al., 2013
			AMPAR trafficking↓ Amplitude of mEPSCs alters the subunit composition of AMPAR↑ Excitatory synaptic currents (Integrin-β3)	Cingolani et al., 2008; Harburger and Calderwood, 2009
LAR-RPTPs	post	NGL-3, Slitrks	Maintains excitatory synapses and dendritic spines, AMPAR trafficking, ↑ AMPAR-mediatedCurrent (LAR)	Dunah et al., 2005
			Excitatory synaptic differentiation ↑ PPF, mEPSCs frequency, ↓ LTP (PTPσ)	Horn et al., 2012; Yim et al., 2013
			Inhibitory synaptic differentiation ↑ PPF, LTP (PTPδ)	Uetani et al., 2000; Yim et al., 2013

pre: pre-synaptic; post: post-synaptic; both: pre- and post-synaptic; ↑: increase; ↓: decrease

## CAMS in neurological disorders

The incidence of neurological disorders is increasing in the human population. For example, in 2006, there were 26.6 million AD patients in the world (Brookmeyer et al., 2007). Autism spectrum disorders (ASD), neurodevelopmental disorders, now affects about 1 % of children (Newschaffer et al., 2007). Most neurological disorders originate as dysfunction of neural circuits, whose function is highly reliant on precisely controlled cell-cell adhesions.

As we discussed above, CAMs, which connect neurons with each other, play a key role in synapse formation and synaptic plasticity. Mounting evidence now connects several neurological disorders with CAMs, as many mutations or aberrant expressions of CAMs are associated with neurological disorders. For example, mutations in Nrxs and NLs are found in ASD patients (Südhof, 2008) and Eph receptor alterations are highly related with AD (Chen et al., 2012). Therefore, research on CAM function will help to provide a better understanding of the mechanisms underlying pathogenic neurological disorders.

### CAMs in autism

ASD are neural development disorders that are often associated with other genetic disorders such as Down syndrome, tuberous sclerosis, and Fragile-X Mental Retardation. ASD are characterized by impairments in social interaction and communication, and stereotypic or repetitive behaviors (Südhof, 2008). ASD alter the connection and organization of nerve cells and their synapses in the brain. Genetic studies have revealed many mutations in CAMs in ASD patients: for example, five ultra-rare structural variants including a predicted splicing mutation have been found in α-Nrx1 gene from 116 Caucasian patients with autism but only one ultra-rare structural variant occurred in controls (Yan et al., 2008). The β-Nrx1 gene has two putative missense structural variants that were detected in four Caucasian patients with autism and not in healthy controls (Feng et al., 2006). On post-synaptic side, two NL genes (NL3 and 4) located on the X-chromosome are associated with autism. Two base pair deletions in NL4 have been found in male autistic patients, resulting in altered interactions with β-Nrxs (Laumonnier et al., 2004). The R451C and R87 W substitutions in the NL3 and NL4 genes, respectively, have been associated with autistic patients (Comoletti et al., 2004; Zhang et al., 2009). This R451C mutation impairs NL3 trafficking, resulting in lower cell surface expression of NL3 and largely reducing β-Nrx1 binding activity.

Mice with a R451C knock-in show increased spatial learning and impairments in social interactions, accompanied by specific increases in inhibitory synaptic transmission (Tabuchi et al., 2007). The R451C mutation is a gain-of-function substitution because NL3 KO mice did not phenocopy any of the phenotypes observed in the knock-in mice (Tabuchi et al., 2007). The largely decreased cell surface expression of NL3 in R451C mutant mice (Comoletti et al., 2004) indicates that the remaining protein must change synaptic function tremendously. Unlike the R451C knock-in mice, a loss-of-function mutation in the mouse NL4 impairs reciprocal social interactions and communication (Jamain et al., 2008). Thus, both NL3 knock-in and NL4 KO mice display autism-like phenotypes, providing partial animal models for this disorder.

Other CAMs are also involved in autism. Genetic studies shows an involvement of cadherin 10 (CDH10) and cadherin 9 (CDH9) in the pathogenesis of autism (Wang et al. 2009). A scan for the IQ discrepancy in autism revealed a unique truncated cadherin, cadherin 13 (CDH13), which has also been suggested as a candidate for autism (Chapman et al. 2011). A genome-wide recurrent *de novo* analysis also includes the CDH13 gene in rare copy-number variations in autism families (Sanders et al. 2011). Cadherin 15 (CDH15) gene has been found in a sporadic patient with autism (Willemsen et al. 2010). Cadherin 8 (CDH8), which presents in the developing human cortex, is reported as an autism susceptibility gene in other recent research (Pagnamenta et al., 2011). A meta-analysis identified several genes close to cadherin with possible links to autism. Protocadherin 10 (PCDH10), which regulates neuronal activity and controls axon outgrowth, is a potential candidate gene for autism (Morrow et al. 2008; Uemura et al. 2007).

CNTN is another family of cell adhesion molecules related to autism. Currently, CNTN4, CNTN5, and CNTN6 are suggested as potential disease genes for autism. First, a deletion at the 5′ end of the CNTN4 gene has been identified in an autism patient (Cottrell et al. 2011). Disruption of the CNTN4 gene causes the 3p deletion syndrome and impairs normal CNS development (Fernandez et al. 2004). Rare copy number variations (CNVs) in CNTN4 have been reported to influence autism susceptibility in Asian populations (Guo et al. 2012). A loss of CNTN5 co-segregated with autism in one family, and one *de novo* CNV and one non-cosegregating inherited CNV in CNTN6 were found in a Utrecht cohort (van Daalen et al. 2011). Current data clearly suggest a link between mutations in different CAMs and autism, but how these mutant proteins give rise to the altered human behavior seen in autism patients is still a mystery.

### CAMs in AD

AD, first defined in 1906 by Alois Alzheimer, is the most common form of dementia, with an increasing risk with age. In Europe, millions of patients suffer from the disease and the numbers of patients are expected to increase dramatically (Di Luca et al. 2011). Multiple neurochemical, neurological, psychological, and physical abnormalities have been reported in AD patients, indicating AD to be a multifactorial disease. It is a slowly progressive disorder, where early memory loss originates from synapse failure before neuron death. Therefore, AD is expected to show a strong relationship with CAMs that play essential roles in intercellular synaptic connections.

One very viable hypothesis suggests that synaptic failure in AD is due to altered synaptic protein composition and function. Ephs and ephrins, which are known to regulate synapse formation and synaptic plasticity, are related with cognitive impairments in AD (Chen et al., 2012). The expression and function of Ephrins and Eph receptors changes in AD patients. In AD model mice, abnormal expression of EphA4 and EphB2 are detected much earlier than the decrease in synaptic proteins and the onset of cognitive decline (Simón et al., 2009), indicating that Eph receptors may act as early stage markers of AD. EphA4 has been reported to colocalize with γ-secretase, the key enzyme that cleaves amyloid precursor protein (APP) to generate Aβ (De Strooper 2003); EphA4 is processed by γ-secretase upon synaptic activity (Inoue et al., 2009). At the synapses, γ-secretase processes EphA4 to generate EphA4-ICD, a short intracellular domain. However, familial mutations in presenilin 1 (PS1) in AD’s patients slow down this process, resulting in a reduced formation of dendritic spines, implying that down-regulated processing of EphA4 may be involved in AD pathogenesis. Moreover, Rac1, which is activated by EphA4-ICD, has been reported to control the activity of the p21-activated kinase (PAK) pathway, leading to memory impairment (Zhao et al., 2006). Indeed, the amount of Rac1 decreases dramatically corresponding with the level of EphA4-ICD in AD patients (Matsui et al., 2012). The processing of the EphB2 receptor is also regulated by γ-secretase and inhibited by familial AD mutations of PS1 (Litterst et al., 2007). The NMDA receptor is phosphorylated by the C-terminal of EphB2, while reduced processing of EphB2 may decrease the cell surface expression of NMDAR, resulting in learning and memory impairment (Xu et al., 2009). The Aβ peptide binds to the extracellular domain of EphB2 and triggers EphB2 degradation in the proteasome, leading to a decrease in surface and total EphB2 in neurons. A lack of EphB2 expression causes neuronal dysfunction and memory impairments through the NMDAR dependent pathway (Cissé et al., 2011). More interestingly, increasing EphB2 level can reverse these impairments.

Many other CAMs are also involved in AD pathogenesis. For example, soluble intercellular adhesion molecule-1 (s-ICAM-1) levels are higher in patients with AD (Rentzos et al., 2005). A genome-wide late-onset AD analysis associates PCDH11X with disease in individuals of European descent from the United States (Carrasquillo et al., 2009). The interactions of Nrxs and NLs not only control the balance between excitatory and inhibitory neurotransmitter release, but they also function in β-amyloid metabolism, suggesting roles in AD (Sindi et al., 2014). Processing of Nrx3β can be altered by several PS1 mutations of the γ-secretase that cause early-onset familial AD (Bot et al., 2011). In hippocampal neurons, the accumulation of Nrx C-terminal fragments is associated with the inhibition of presenilin/γ-secretase (Saura et al., 2011). N-cadherin enhances APP dimerization, while its C-terminal fragment accelerates Aβ and causes synapse damage (Asada-Utsugi et al., 2011; Andreyeva et al., 2012). β-catenin mediates the structural changes associated with memory formation, suggesting a role in memory impairment (Maguschak and Ressler 2012). Nectin-1 serves as a substrate for PS/γ-secretase-like intramembrane proteolytic activity (Kim et al., 2002). Nectin-3 is also cleaved by intramembrane PS1/γ-secretase (Kim et al., 2011). Therefore, altered functions in various CAMs contribute to the pathogenesis of AD, although the precise underlying mechanism is still unknown.

### CAMs in other diseases

CAMs are involved in many other neurological diseases beyond autism and AD. Schizophrenia, a mental disorder characterized by social withdrawal, paranoid delusions, and hallucinations, is associated with abnormal expression and function of CAMs. Embryonic NCAM dysfunction was linked with schizophrenia more than 20 years ago (Conrad and Scheibel 1987). Unlike the case of ASD patients (Plioplys et al., 1990), NCAM levels in serum and cerebrospinal fluid (CSF) increase in schizophrenic patients (Lyons et al., 1988; Poltorak et al., 1996). Interestingly, the hippocampus of schizophrenic patients shows a reduction of polysialylated NCAM (Barbeau et al., 1995). An increase in the cytosolic isoform of NCAM has also been observed in the hippocampus of schizophrenia patients (Vawter et al., 1998a). The ratio of NCAM/synaptic proteins is also changed in some cases, indicating alterations in mature/immature synapses. For example, an increase in the cytosolic NCAM/synaptophysin ratio was demonstrated in the hippocampus of schizophrenia patients (Vawter et al., 1999). Similarly, the cingulate cortex of schizophrenics also showed elevated NCAM/synaptophysin ratios (Honer et al., 1997). Much research in autism and mental retardation has implicated Nrxs in schizophrenia (Kirov et al., 2008). Whole-genome analysis conducted in 2008 identified a deletion in two affected siblings that disrupted Nrx1 (Kirov et al., 2008). Nrx1α exonic deletions have since been found in three patients with paranoid-type schizophrenia (Vrijenhoek et al., 2008). A later study of 2977 schizophrenia patients and 33746 controls examined Nrx1 for copy number variants (CNVs) and identified 66 deletions and 5 duplications in NRXN1 from the patients, confirming that Nrx1 is a risk gene for schizophrenia (Rujescu et al., 2009). Bipolar (BP) disorder is another neuropsychiatric disorder that is related to CAMs. Recently, a genome-wide association scan of BP 1 disorder patients identified some single nucleotide polymorphisms (SNPs) in close vicinity to cadherin 7 (CDH7) (Sklar et al., 2008; Soronen et al., 2010). In 2010, PCDH9 was recognized as one of the target genes of β-catenin for schizophrenia and BP disorders (Pedrosa et al., 2010). Susceptibility to BP disorder was also found associated with FAT, a cadherin gene, in four independent cohorts (Blair et al., 2006).

The cytosolic NCAM isoform (cN-CAM) undergoes a tremendous reduction in BP disorder (−140%) in hippocampal tissue (Vawter 2000). Quantitative Western blot analysis revealed that cytosolic NCAM protein and mRNA levels increased in the hippocampus and prefrontal cortex in BP disorder patients (Vawter et al., 1998b). Interestingly, NCAM infusion reduced astrocyte division, while BP disorder decreased glia numbers (Krushel et al., 1995; Ongür et al., 1998). Thus, different NCAM isoforms may play multiple roles in different brain regions in BP disorder patients.

Although only some superficial evidence that try to reveal the underlying mechanisms how CAMs are involved in neurological diseases pathogenesis can be observed, two points are seemed quite certain: on one hand, many CAMs could contribute in one neurological disease; on the other hand, one altered CAM could result in several neurological disorders, both in either separate or in cooperative way. Thus no ‘leading’ protein represents a common pathway for each of the neurological diseases, it is more likely that ‘many hands make light work’ in nature. Therefore more investments are required to deepen our understanding of the mechanisms of the molecular regulation of synapses.

## Summary

Neurons communicate via synaptic connection mostly mediated by precisely-controlled intercellular interactions. CAMs are involved in all stages of synapse formation and stabilization, providing ‘bridges’ between pre- and post-synaptic sites. At present, mounting evidence clearly indicates that no single pair of CAMs is necessary or sufficient for the organization of synapse developments from initiation to maturation, indicating overlapping or redundant functions of CAMs. The diversity of the isoforms and functions of CAMs may contribute to the complexity of neuronal network. Abnormalities in CAMs often cause neurological diseases.

The involvement of CAMs in synaptogenesis and synaptic connection is now well accepted. However, it remains unclear that actually how many and which proteins are involved in the process of synaptogenesis, how these distinct CAMs contribute to the specific synapse sybtypes and functions and how the CAMs cooperate together. At present, in view of the large variety of synapses in the brain, the number of known CAMs is surprisingly low. It is natural to speculate that more proteins are involved in this process, perhaps even some ‘old’ molecules with recognized activities in other aspects of functions. For instance, SNAP25, one component of the SNARE complex required for synaptic exocytosis, regulates dendritic spine maturation and function through its expression level (Tomasoni et al., 2013). Complexin2, a key regulator of neurotransmitter release, also has a role in synaptogenesis (Lee et al., 2005). A deficit in Munc18 or Munc13 also reduces the outgrowth speed of neurite at the early stage of development (Broeke et al., 2010). Thrombospondins (TSPs), proteins known in angiogenesis and many immune regulations, promote neuronal synaptogenesis *in vitro* and *in vivo* (Christopherson et al., 2005). Although these proteins may interfere with CAM-mediated intercellular interactions or receptors trafficking rather than function in synaptogenesis directly and make the cases even more complicate. Together, determining the proteins that take part in synapse developments is one of the highest priorities in neuroscience research. Clearly, large-scale screening with better technology or improved design might be an inspiring way to uncover additional new CAMs, or identify already known synaptic proteins such as CAMs, to provide a better understanding of the physiology and pathology occurring during synapse development.

## Abbreviations

AChR: acetylcholine receptor
AD: Alzheimer’s disease
CAMs: cell adhesion molecules
CNTN: contactins
Cdk5: cyclin-dependent kinase 5
FGF: fibroblast growth factor
GKAP: guanylate kinase-associated protein
KO: knockout
LAR-RPTPs: leukocyte antigen-related receptor protein tyrosine phosphatases
LRRTMs: leucine-rich repeat transmembrane neuronal proteins
LTD: long-term depression
MAPK: mitogen-activated protein kinase
NCAM: neural cell adhesion molecule
NLs: neuroligins
Nrxs: neurexins
PSD: post-synaptic density
SAM: sterile alpha motif
SALMs: synaptic adhesion-like molecules
SynCAM: synaptic cell adhesion molecules
